# Octreotide in Palliative Treatment of Unresectable Cholangiocarcinoma: Is it Effective for Disease Stabilization?

**DOI:** 10.4137/cmo.s677

**Published:** 2008-04-24

**Authors:** Kiki Pistevou-Gompaki, Nikos Eleftheriadis, Christos Papaloukas, Damianos Eleftheriadis

**Affiliations:** Department of Radiation Oncology, Aristotle’s University of Thessaloniki, Greece

**Keywords:** octreotide LAR, cholangiocarcinoma, Klatskin tumor, palliative treatment

## Letter to the Editor

Cholangiocarcinoma is a dismal tumor, difficult to treat and in the majority of cases surgery is the only available method of cure, however only in early stages.^1^ In advanced cases (turmors Klatskin III and IV) palliative therapy with endoscopic drainage of bile ducts is the only effective and acceptable method of treatment.^2^ Adjuvant palliative therapies either with radiotherapy or chemotherapy in order to stabilize the disease gave controversial results and in some aspect unacceptable.^1^–^2^ The need for new treatment to stabilize the disease, increase survival with acceptable quality of life is urgent.

Somatostatin and its long-acting analogues have been successfully used in symptom control in patients with advanced neuroendocrine gastrointestinal tumours.^3^,^4^ Moreover, octreotide, a somatostatin analogue, showed significant efficacy for the management of hepatocellular carcinoma in many studies and reviews.^5^ Pistevou-Gombaki et al.^6^,^7^ also reported a positive experience in liver metastases from non-neuroendocrine tumors with the use of octreotide LAR.

A possible antitumor mechanism of octreotide is a stimulatory effect on Kupffer cells, induction of apoptosis or other antiproliferative actions, inhibition of proliferation, which have been suggested but not proved.^3^,^4^

The aim of the present study is to evaluate the role, if any, of octreotide in palliative treatment of end-stage, inoperable, cholangiocarcinomas (Klatskin III and IV tumors), taking into account the above-mentioned positive experience in relation to the absence of clear data and efficacy of any available treatments in end-stage cholangiocarcinomas.

We report on two male patients (A, B) 63- and 81- years-old, with obstructive icterus, due to advanced cholangiocarcinoma stage Klatskin III and IV respectively and liver metastases in patient A, who were palliatively treated by long acting octreotide IM (octreotide LAR) monthly, according to schema published previously,^7^ in combination with successful endoscopic bile duct drainage.

In both patients Klatskin tumor was demonstrated, in first instance by abdominal echosonography and computed-tomography (CT), and thereafter by magnetic resonance imaging (MRI) and magnetic retrograde cholangiopancreatography (MRCP). Operation was excluded either due to advanced disease in patient A (liver metastases) and due to advanced age in patient B (81 years-old). Liver transplantation was also excluded due to advanced age and disease.

Clinical examination revealed severe liver mass in both patients. Laboratory examination showed high bilirubin levels up to 30 mg/dl direct 20 mg/dl indirect 10 mg/dl, increased γ-GT, SGPT, ALF and highly increased tumor markers (CEA >1000 and CA19–9 >1000 mg/dl) in both patients. CRP was also elevated in both patients without however any other signs of overt cholangitis. In MRCP gallbladder was excluded in both patients due to infiltration of cystic duct by the tumor.

According to clinicolaboratoty results of these cases, the patients were urgently treated by ERCP, schicterotomy and successful metallic stent placement in order to drain the bile ducts. Both patients were discharged and bilirubin normalized in a few days. Radiotherapy or chemotherapy was refused from both patients after having explained the risk-benefit ratio. The patients complained for general symptoms attributed to Klatksin tumors, including right upper abdominal pain, loss of appetite, malaise and weight loss.

At first instance all patients were under supportive therapy with common analgesics and enteral nutrition, upon indication, with mild improvement of right upper abdominal pain. A month after metallic stent placement and successful bile duct drainage, the right upper abdominal pain, attributed to liver tumor, deteriorated and produced severe morbidity, which was not responding to further increase of analgesics. Laboratory examination showed normal bilirubin levels, which remained normal during follow-up six months after stent placement.

At that time and in combination with the absence of clear data to support any benefit from any therapy in these debilitating patients, in order to stabilize the disease, it was decided to further treat the patients with 20 mg long acting octreotide IM (octreotide LAR), once the first day, octreotide SC 0,5 mgX3/daily, days 2–14 and then 20 mg long acting octreotide IM montly, as in previous studies in patients with symptomatic liver metastases.^6^,^7^

The follow-up was done with clinicolaboratory examination and abdominal ultrasonography before and at one-month intervals after the initiation of octreotide therapy. The evaluation of the pain related to cholangiocarcinoma was performed at baseline and monthly after initiation of octreotide-treatment, using the visual analogue scale (VAS). The palliative effect was also assessed every month using the analgesic intake scale (AIS) according to WHO (0 = no analgesics; 1 = NSAID; 2 = weak opioids; 3 = morphine).

One month after the administration of octreotide, the right upper abdominal pain was in general terms decreased. In fact, both patients reported moderate to excellent subjective improvement with 3 and 4 points decrease of VAS score in patient A and B respectively. An interesting point of the study was that, the local pain at the right upper abdomen relapsed when octreotide was temporarily stopped or at the end of every month just before the next injection of octreotide LAR and reduced again soon after the initiation of octreotide. Furthermore, one month after octreotide was started the AIS was significantly reduced (from 3 to one in patient A and from 3 to 2 and then to one in patient B) and remained in these levels during the six-months follow-up.

As far as the side effects are concerned octreotide was generally well-tolerated. Slight hyperglycemia was reported in both patients treated by specific diet. No other severe side effects due to octreotide-treatment were reported.

**Figure 1:**
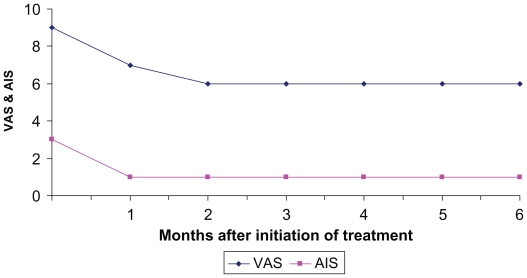
Time course of VAS and AIS values in pt A after octreotide administration.

**Figure 2:**
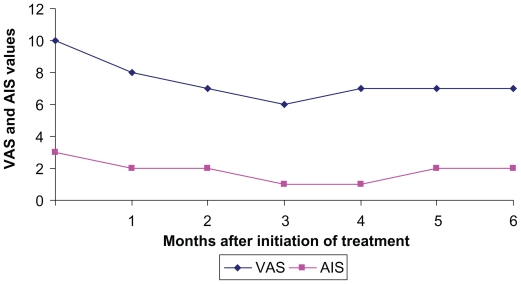
Time course of VAS and AIS values in pt B after octreotide administration.

During follow-up, six months after the initiation of octreotide therapy, both patients were in good general condition, local pain improvement was still reported, no analgesics were necessary and both patients were still alive without icterus. Although endoscopic metallic stent placement was successful for bile duct drainage, abdominal pain attributed to tumor growth persisted and even increased, soon after stent placement. In view of this evolution, we decided to further treat the patients with octreotide LAR, which resulted in good pain control and reduce of analgesics. The most important is that the values of VAS and AIS remained stable after the first month of improvement, as it is shown in Figures 1 and 2. Kapadia commenting on the improvement of quality of life after the administration of octreotide in patients with hepatocellular carcinoma, reported that the possible mechanism of this should be the diminishing of the effects of various humoral agents and/or cytokines released from the tumor.^8^

To our knowledge this is the first study of the palliative use of octreotide in end-stage cholangiocarcinoma, although the inhibitory effects of octreotide on cholangiocarcinoma cell lines have been investigated in vitro and in vivo with positive results.^9^ Zhao B et al.^9^ reported that octreotide inhibits the proliferation of cholangiocarcinoma cells through G0/G1 cell cycle arrest. It seems that a potential new therapeutic approach for cholangiocarcinoma has been investigated in the present study. However, we did not have control group and the small number of patients made the evaluation of our results more difficult.

We consider the use of octreotide potentially effective in disease stabilization and a good alternative in palliative treatment of end stage cholangiocarcinoma with minimum or absence of side effects. The positive results of the present study encourage further randomized controlled trials of octreotide in the treatment of end-stage cholangiocarcinomas.
